# Identification of Rotavirus Genotypes in Children under Five Years in the United Arab Emirates Using Nanopore Sequencing Technology

**DOI:** 10.1002/jmv.70056

**Published:** 2024-11-08

**Authors:** Junu A. George, Farah Al‐Marzooq, Hassib Narchi, Ahmed R. Alsuwaidi

**Affiliations:** ^1^ Department of Pediatrics, College of Medicine and Health Sciences UAE University Al‐Ain UAE; ^2^ Department of Microbiology and Immunology, College of Medicine and Health Sciences UAE University Al‐Ain UAE

**Keywords:** genotypes, nanopore sequencing, PCR, rotavirus, UAE

## Abstract

Group A rotaviruses (RVA) remain a principal cause of childhood diarrhea in the UAE, despite universal vaccine use. Monitoring genetic diversity is important for identifying prevalent genotypes and escape mutants. Although real‐time polymerase chain reaction (RT‐PCR) is widely used for RVA genotyping, it may not detect some new strains. This study evaluates nanopore sequencing and RT‐PCR for RVA genotyping. Thirty‐three RVA strains from children under 5 years presenting with diarrhea were genotyped using both methods. Thirteen strains were genotyped by RT‐PCR and confirmed by nanopore sequencing. Fifteen strains were genotyped by nanopore method alone. Most PCR‐genotyped strains (56%) had the VP7 G9 genotype, with G3 in five strains and G12 in two. For VP4, P8 (*n* = 8) and P4 (*n* = 7) were dominant. The most frequent combinations were G9P[8] (31%) and G9P[4] (25%). Nanopore sequencing of 28 strains revealed G3P[8] (29%) as the most prevalent, followed by G8P[8] (18%). G9P[8] and G2P[4] were present in 14% of samples with G12P[6] being the rarest (7%). Other combinations were detected in 4% the specimens with one nontypeable. Nanopore sequencing was superior to PCR in identifying diverse and emerging genotypes like G8P[8]. This method may enhance surveillance studies and guide preventive measures for RVA gastroenteritis.

## 1 Introduction

Group A rotaviruses (RVA) are important cause of acute gastroenteritis in children under 5 years of age [1]. In 2009, the World Health Organization (WHO) recommended all national immunization programs to include rotavirus vaccination for infants [2]. To date, more than 110 countries have introduced rotavirus vaccines into their immunization programs with evidence of significant reductions in rotavirus and acute gastroenteritis hospitalizations particularly in countries with higher vaccine coverage [3, 4]. Internationally available and WHO‐prequalified RVA vaccines include RotaTeq (a reassorted bovine‐human pentavalent vaccine by Merck Vaccines), Rotarix (a monovalent vaccine derived from a human rotavirus strain by GlaxoSmithKline Biologicals), Rotavac (a monovalent vaccine featuring a naturally occurring bovine‐human reassortant neonatal G9P, by Bharat Biotech), and RotaSiil (a reassorted pentavalent bovine‐human vaccine with a human G1, G2, G3, and G4 bovine UK G6P[5] backbone, by the Serum Institute of India). Although the approved vaccines have been shown to provide cross protection to heterotypic strains, it is important to continuously monitor the diversity of circulating RVA strains after vaccines introduction into the national immunization programs [5].

Rotavirus genome consists of 11 double‐stranded RNA (dsRNA) segments. Gene segments 9 and 4 have the highest epidemiological value, since they encode neutralizing RVA antigens G (VP7) and P (VP4), and determines the G/P viral genotype [6]. More than 60 G/P combinations have been identified in human infections with genotypes G1P[8], G2P[4], G3P[8], G4P[8], G9P[8] and G12P[8] being the most prevalent circulating genotypes worldwide [7].

The WHO has been carrying out a rotavirus surveillance program since 2008 aiming to generate data for development, implementation, and monitoring of rotavirus vaccination programs. Initially, the program involved the use of polymerase chain reaction (PCR) methods including specific primers to identify different G and P genotypes. This approach required constant review and optimization of primers as more and more nontypeable isolates emerged. The WHO currently recommends all nontypeable isolates be sent to an appropriate reference laboratory for sequencing [8].

The nanopore based sequencing (NPS) is one of the newly emerging sequencing methods. It was developed by Oxford Nanopore Technologies, United Kingdom. The method, used for genotyping many viruses, has several advantages including low start‐up costs compared to other high‐throughput platforms, ability to obtain long reads allowing sequencing up to several thousand bases from individual RNA and DNA molecules, and rapid turnaround time. In one study, NPS allowed rapid analysis of highly multiplexed targets across large number of samples with a potential cost reduction by up to 200 times compared to Sanger sequencing [9]. Although base‐calling accuracy of the NPS method is less compared to other platforms, it is still sufficient for phylogenetic studies. Phylogeny of segmented viruses using NPS is mostly carried after amplification with universal primers, which is known to introduce amplification errors [10, 11, 12]. The ideal template for NPS sequencing is double stranded DNA (dsDNA), but there are attempts to standardize the protocol for genotyping rotavirus which is dsRNA virus for large scale surveillance studies [13].

In the United Arab Emirates (UAE), only Rotarix and RotaTeq vaccines have received licensure from the Ministry of Health. In June 2013, the UAE integrated the Rotarix vaccine into its national immunization program, with a two‐dose schedule, administered at 2 and 4 months of age [14]. Despite universal use and high vaccine coverage (> 90%), rotavirus remains the most prevalent pathogen in young children with diarrhea [15, 16]. This may be attributed to UAE's diverse population, with many residents originating from various countries, where different host, maternal and environmental factors could influence the vaccine response [17]. Additionally, while rotavirus vaccine is effective in protecting against severe gastroenteritis, it does not completely prevent infection. Moreover, the RVA vaccines target common strains, and emerging ones may evade the vaccine‐induced immune response. Thus, it is vital to continuously assess the long‐term impact of vaccination on circulating RVA strains. Whole‐genome longitudinal surveillance studies are ideal tools to evaluate potential RVA vaccine‐induced strain changes.

In this study, we utilized an optimized protocol for genotyping RVA using nanopore sequencing technology, aiming to explore the genetic diversity of rotavirus strains isolated from young children with diarrhea in the UAE. This study is the first from the UAE to describe RVA genotypes after the introduction of rotavirus vaccines into the national immunization program. The findings will provide valuable insights for future rotavirus vaccine strategies in the UAE and highlight the significance of continuous global monitoring of the circulating RVA strains.

## 2 Methods

### 2.1 Selection of Samples for Genotyping

**Figure 1:**
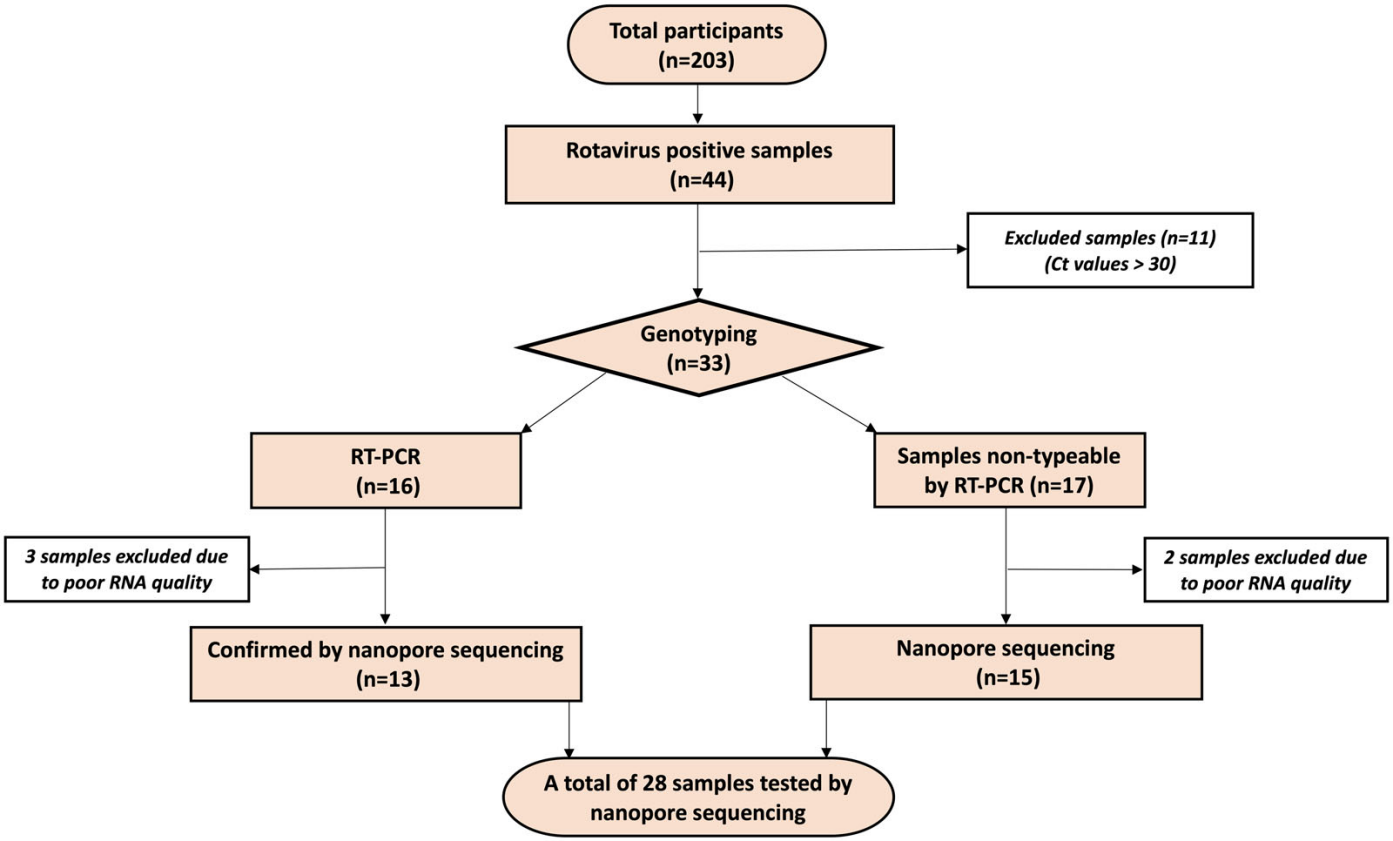
Flowchart representing the selection of samples for RT‐PCR and sequencing.

A total of 203 fecal samples were collected between December 2017 and April 2019 from children less than 5 years of age who presented to Tawam and Al Ain hospitals in Al Ain city, UAE with diarrhea as described previously [16]. Initial results from Allplex™ Gastrointestinal Full Panel Assay revealed that 21.7% (*n* = 44) of fecal samples were positive for rotavirus. A total of 33 samples were selected for genotyping and 11 samples were excluded due to low viral load as reflected in the high threshold cycle values (Ct > 30). Vaccination history was recorded for the selected samples. Figure 1 represents selection criteria of the samples and workflow for genotyping by qRT‐PCR and nanopore sequencing. As shown in Figure 1, a total of 28 samples were finally sequenced using nanopore technology to identify rotavirus genotypes.

### 2.2 RNA Extraction

Total RNA was extracted using stool total RNA purification kit (Norgen, Biotek Corp, ON, Canada) according to the manufacturer's instructions. The kit isolates total RNA with minimal amounts of DNA contamination. Briefly, 200 mg of stool was lysed in a bead tube using 1 mL of lysis buffer, vortexed for 5 min before centrifuging at 14 000 g for 3 min to remove solid fecal matter. Then, 600 µL of the supernatant was transferred to a clean tube and mixed with an equal volume of 70% ethanol. Samples were then loaded onto a spin column with collection tube and centrifuged for 1 min at 14 000 g. After washing the column twice with washing buffer, the column was transferred onto a fresh elution tube. The final elution of RNA was performed with 75 µL of the elution buffer.

### 2.3 cDNA Synthesis and Genotyping By Taqman qRT‐PCR

Reverse‐transcription for cDNA synthesis was carried out in a final volume of 50 µL using MMLV RT kit (Promega, USA). Initially, 500 ng RNA was denatured by incubation at 95℃ for 3 min. The mixture was immediately snap chilled on ice followed by the addition of 0.7 µL random hexamer (350 ng), and 5 μL of 10 mM dNTPs which was incubated at 70℃ for 5 min. This mixture was again snap chilled for 3 min. 10 μL of 5× RT buffer, 40 U RNase Inhibitor and 200 U MMLV‐RT (Promega, USA) were made up to the required volume using deionized water and kept at 37℃ for 1 h. Completed RT reactions were stored at −20℃ until further analysis.

Genotype specific primers and probes of VP7 and VP4 were selected as mentioned elsewhere [18]. A typical 20‐μl real‐time PCR amplification reaction contained 10 μL of 2 × TaqMan Universal Master mix, 1.5 μL (10 nM) of the appropriate forward and reverse primer(s) and 1 μL of probe (s) and 4 μL of cDNA RT reaction. The following rotavirus isolates were selected as positive controls for each genotype: Wa (G1P[8]), DS‐1 (G2P[4]), AU‐1 (G3P[9]), ST3 (G4P[6]), 69 M (G8P[10]), 116E (G9P[11]), I‐321 (G10P[11]), L26 (G12P[4].

### 2.4 Sample Preparation for Viral Whole Genome Sequencing

#### 2.4.1 Double Stranded RNA Purification Using Lithium Chloride

Total extracted RNA contained host and microflora RNA along with viral genomic RNA. To enrich Rotavirus dsRNA, lithium chloride (LiCl) precipitation method was devised. Briefly, the eluted 75 µL of total RNA was incubated for 8 h at 4℃ with 2 M LiCl (final concentration). The supernatant obtained after centrifugation and dsRNA pellet was recovered by centrifugation (14 000 rpm for 10 min at 4℃). The pellet was washed in 70% ethanol and re‐suspended in 20 µL of Rnase/DNase free water. Samples with low RNA quality (260/280 ratio) were excluded from further analysis. A total of 28 samples were found suitable for downstream processing.

#### 2.4.2 Double‐Stranded cDNA Synthesis for Sequencing

Purified RNA was reverse‐transcribed using the Maxima H‐minus double stranded cDNA synthesis kit (Thermo scientific, USA) according to manufacturer's instructions. Briefly, to generate the first cDNA strand, 12 µL of purified dsRNA was incubated for 3 min at 95℃ before adding 2 µL of random hexamer (provided with the kit). After gentle pipetting, the mixture was incubated for 5 min at 65℃. The reaction was snap cooled on ice for 1 min, then centrifuged for 30 s. Subsequently, 5 µl of 4× First Strand Reaction Mix and 1 µL of First Strand Enzyme Mix were added, mixed by slowly pipetting, then incubated on a Qantarus Q‐Cycler (Quanta Biotech Ltd, KT14 7JX, UK) at 25℃ (10 min), 50℃ (30 min) and 85℃ (5 min) to stop the reaction. To produce the second cDNA strand, the entire first strand cDNA reaction mixture (20 µL) was combined with molecular grade water (55 µL) (Sigma Aldrich, Arklow, Ireland), 5× second strand reaction mix (20 µL) and second strand enzyme mix (5 µL). The entire volume (100 µl) was mixed by pipetting and incubated at 16× for 60 min. The reaction was stopped by adding 6 µL of 0.5 M EDTA (pH 8.0). Residual RNA was removed by RNase1 treatment (supplied in Maxima H Minus Double‐Stranded cDNA Synthesis Kit). The double‐stranded cDNA reaction was purified using phenol‐chloroform method. Quantity and quality of cDNA was assessed using Qubit double stranded DNA HS (high sensitivity) assay (Invitrogen, USA).

### 2.5 Nanopore Library Preparation and Sequencing

Ends of dsDNA prepared as described above were blunted using the Oxford Nanopore 1D ligation sequencing kit (SQL‐LSK 109). The blunt DNA was ligated with barcodes using the native barcoding kits (EXP‐NBD 104 and EXP‐NBD 114), and enzyme T4 ligase. After barcoding, DNA was purified using AMPure XP beads (Beckman Coulter). Adapter ligation done for 12 barcoded samples were pooled using Adapter mix II (AMII), NEB next Ultra II ligation master mix and NEB next ligation enhancer (New England Biolabs, UK). Ligated products were cleaned‐up using the AMPure XP beads and short fragment buffer. The adapter ligated samples were eluted in 15 µL of elution buffer. A library mix containing 12 µL of the DNA, 25.5 µL of the loading beads and 37.5 µL of the sequencing buffer was prepared and loaded on a R9.4.1 flow cell (FLO‐MIN106) and sequenced for 12–24 h (Oxford Nanopore, UK).

### 2.6 Genome Alignment and Phylogenetic Analysis

The MinION generated sequences were base called using MinKNOW software (version 19). FASTQ sequence files were converted to FASTA files using Geneious prime software. All the reads that passed QC test (score > 7) were then mapped to the corresponding VP7 and VP4 references using Minimap2 [19]. The consensus sequences were compared with those available in the GenBank database using the Standard Nucleotide BLAST software package (http://www.ncbi.nlm.nih.gov/BLAST). Sequences were aligned with relevant sequences downloaded from GenBank using clustalW. Phylogenetic analysis was performed using Molecular Evolutionary Genetic Analysis (MEGA11) software. Genetic distances were calculated with the Tamura three‐parameter at a nucleotide level, and phylogenetic trees were constructed by the Neighbor‐joining Method with 1000 bootstrap replicates.

### 2.7 Data Availability

Reference and potential candidate vaccine strains were retrieved from GenBank, including Rotarix (GenBank accession number: JN849114), and RotaTeq (GenBank accession numbers: GU565057, GU565068). The sequences generated from this study were deposited in the GenBank database, including VP4 sequences submitted under GenBank accession numbers ON791605‐ON791612, while VP7 sequences were submitted under GenBank accession numbers ON456079 ‐ ON456085; ON500504 ‐ ON500513.

## 3 Results

### 3.1 Distribution of Circulating Rotavirus G/P Genotypes

#### 3.1.1 Genotypes Identified By qRT‐PCR

**Figure 2:**
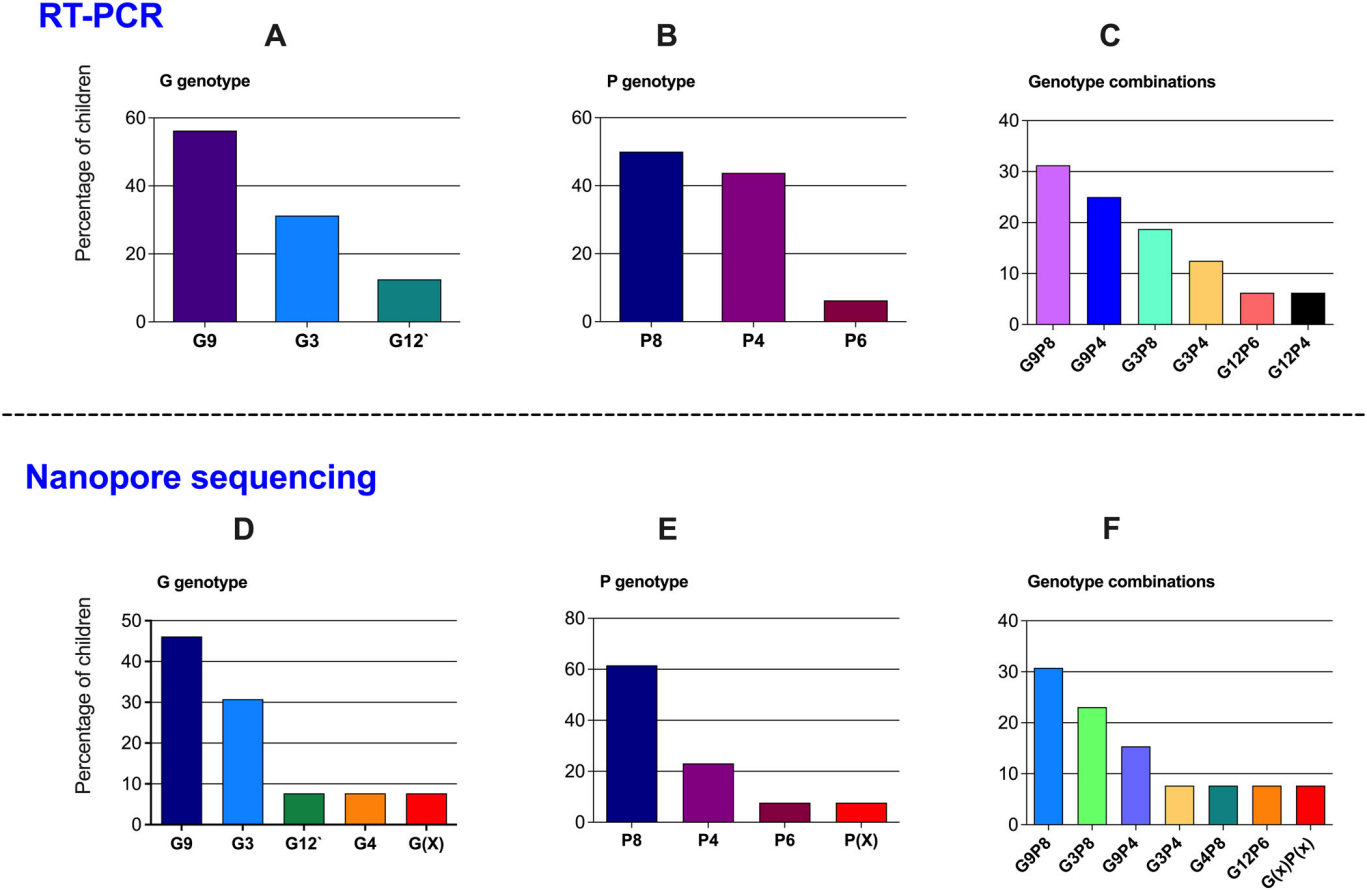
Genotypes identified by RT‐PCR and nanopore sequencing from the same samples. *Upper panel*: (A) G (VP7) typing, (B) P (VP4) typing, and (C) genotypes combination of the rotavirus strains typed by RT‐PCR (*n* = 16, two samples were excluded because of poor RNA quality). *Lower panel*: (D) G (VP7) typing, (E) P (VP7) typing, and (F) genotypes combination by nanopore sequencing (*n* = 13, three samples were excluded because of poor RNA quality). The graph presents percentage of rotavirus strains of G and P genotypes detected in the children; (X: nontypeable).

Simplex TaqMan based qRT‐PCR was used to genotype the isolates based on VP7 (G) and VP4 (P) genes. Genotyping with qRT‐PCR was conclusive for 16 samples and genotypes of the remaining 17 samples could not be determined by PCR method. The majority of isolates (*n* = 9, 56%,) were found to carry VP7 gene of G9 genotype, whereas five isolates (31%) were of G3 genotype. G12 genotype was the rarest, being present only in two isolates (12.5%), Figure 2A. A total of three different VP4 genotypes were detected in the samples (P4, P6, and P8). P8 was the most prevalent genotype present in half of the isolates followed by P4 which was present in seven isolates (44%) and P6 genotype was present only in one isolate (6%), Figure 2B. Combinations of VP7 and VP4 genotypes led to the detection of G9P8 and G9P4 as the most common genotypes, Figure 2C.

#### 3.1.2 Comparison of Genotyping By qRT‐PCR and Nanopore Methods

To confirm the genotyping results obtained by qRT‐PCR, the above 16 samples were further tested by nanopore method; 3 samples were excluded from nanopore sequencing due to low RNA quality as outlined in Figure 1. The results of nanopore sequencing were in agreement with that obtained by qRT‐PCR except for three samples. In case of VP7, four genotypes were identified using nanopore sequencing (G3, G4, G9 and G12). Among them, G9 was the predominant type (6/13; 46%). Other genotypes were in the following order: G3 (4/13; 30.7%), G12 (1/13; 7.6%), and G4 (1/13; 7.6%). G4 genotype was not detected by the RT‐PCR method, but was discovered by nanopore sequencing. Among the 13 samples subjected to nanopore sequencing, the genotype of one isolate could not be determined, Figure 2D. In case of VP4 gene, three P genotypes were identified and P8 was the most common genotype (8/13; 61.5%). Genotype P4 was detected in 3 of the 13 samples whereas P6 was detected in only one sample. In the sample from which G genotype could not be determined, P genotype also could not be attributed to any known genotype, Figure 2E. Thus, among 13 samples, G and P genotypes were identified for 12 samples only. Six different combinations of G and P genotypes were observed. The dominant combination G9P8 was present in 4 samples followed by G3P8 being present in 3 samples out of 12 for which data is available. G9P4 was detected in 2 cases with sequencing, whereas it was detected in 3 samples by qRT‐PCR. The combination of G3P4, G4P8 and G12P6 were also detected in one sample each, Figure 2F.

### 3.2 Genotyping of Samples Failed at qRT‐PCR but were Detectable by Nanopore Sequencing

**Figure 3:**
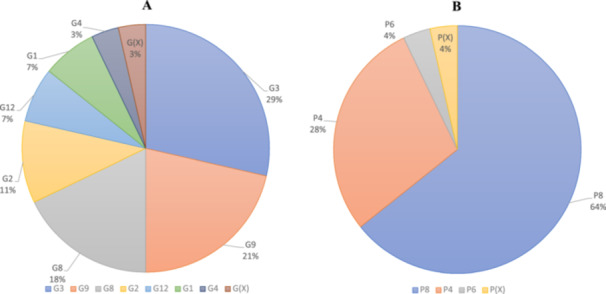
Frequency of (A) VP7 and (B) Frequency of VP4 genotypes in the samples as identified by nanopore sequencing (*n* = 28).

**Figure 4:**
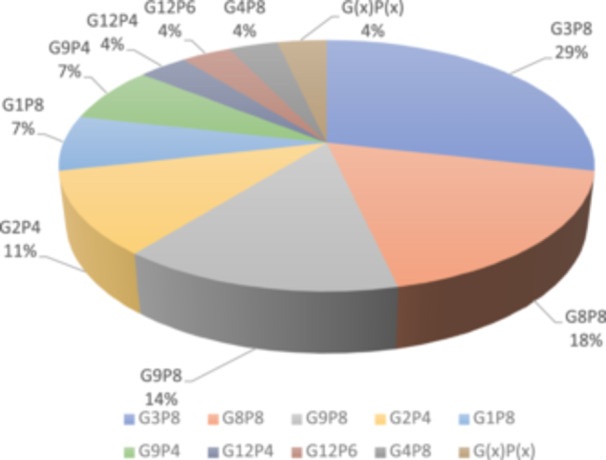
Distribution of G/P genotype combinations in the studied samples.

qRT‐PCR could not identify the RVA genotypes in 17 samples; thus, they were subjected to nanopore sequencing which was successful in detecting RVA genotypes in only 15 samples as two were excluded due to poor RNA quality. Thereby, sequence information of a total of 28 samples were obtained using nanopore sequencing. Bioinformatic analysis revealed the following genotypes of RVA. Among VP7 genotypes, G3 was most the predominant type (29%), followed by G9 (21%) and G8 (18%). Genotypes G12 and G1 were present in 7% of cases. Genotypes G2 and G4 were present in 11% and 4% of cases respectively, Figure 3A. In case of VP4 gene, three P‐genotypes were detected. The frequency of P8 type was around 64%, followed by P4 which was detected in 29% cases. P6 was detected only in 4% cases, Figure 3B. As described above, one sample could not be typed for either G or P. Altogether, 9 different genotype combinations were detected in 28 sequenced cases. The dominant G‐P combination was G3P8 (29% of the samples), followed by G8P8 (18%). G9P8 (14%) and G2P4 (11%). G1P8 and G9P4 were the rarest with only 7% samples having that combination. Further, G12P6, G12P4, and G4P8 were detected in 4% of total specimens, Figure 4.

**Figure 5:**
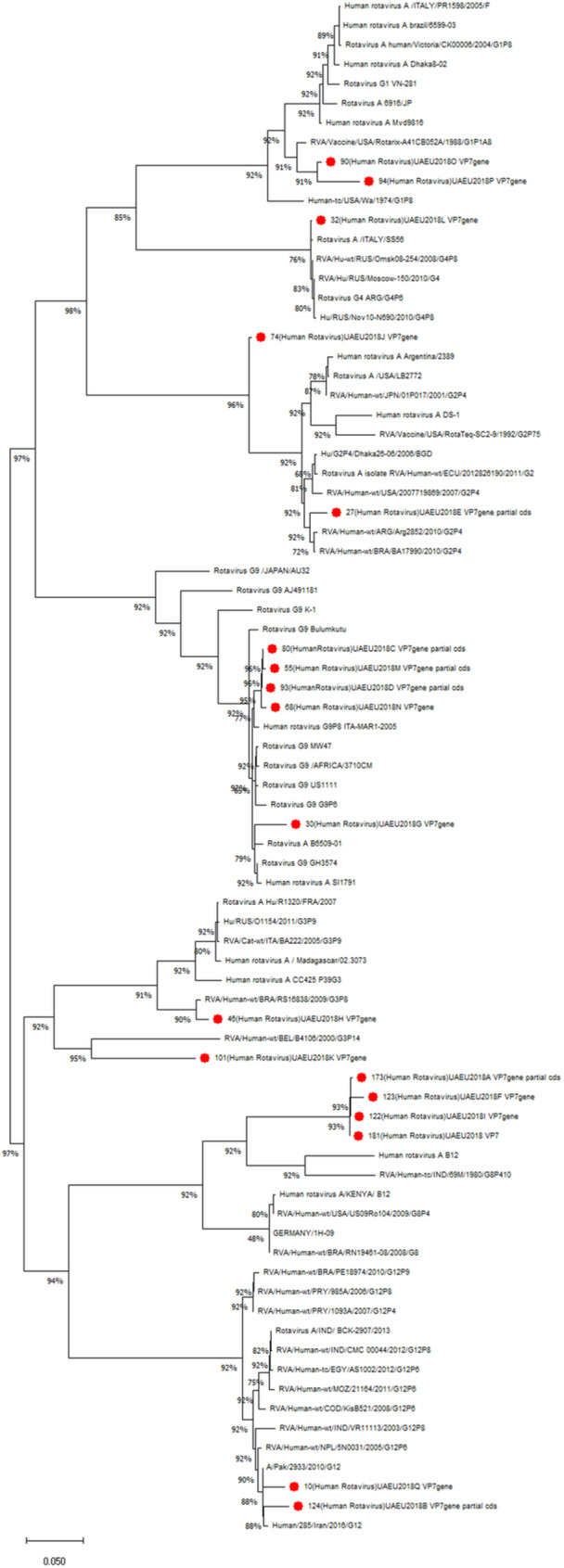
Phylogenetic tree of human rotavirus A strains based on partial VP7 nucleotide sequences. Bootstrap confidence limits are shown at each node; values less than 50 are not shown. Sequences from this study are marked by red color dot.

**Figure 6:**
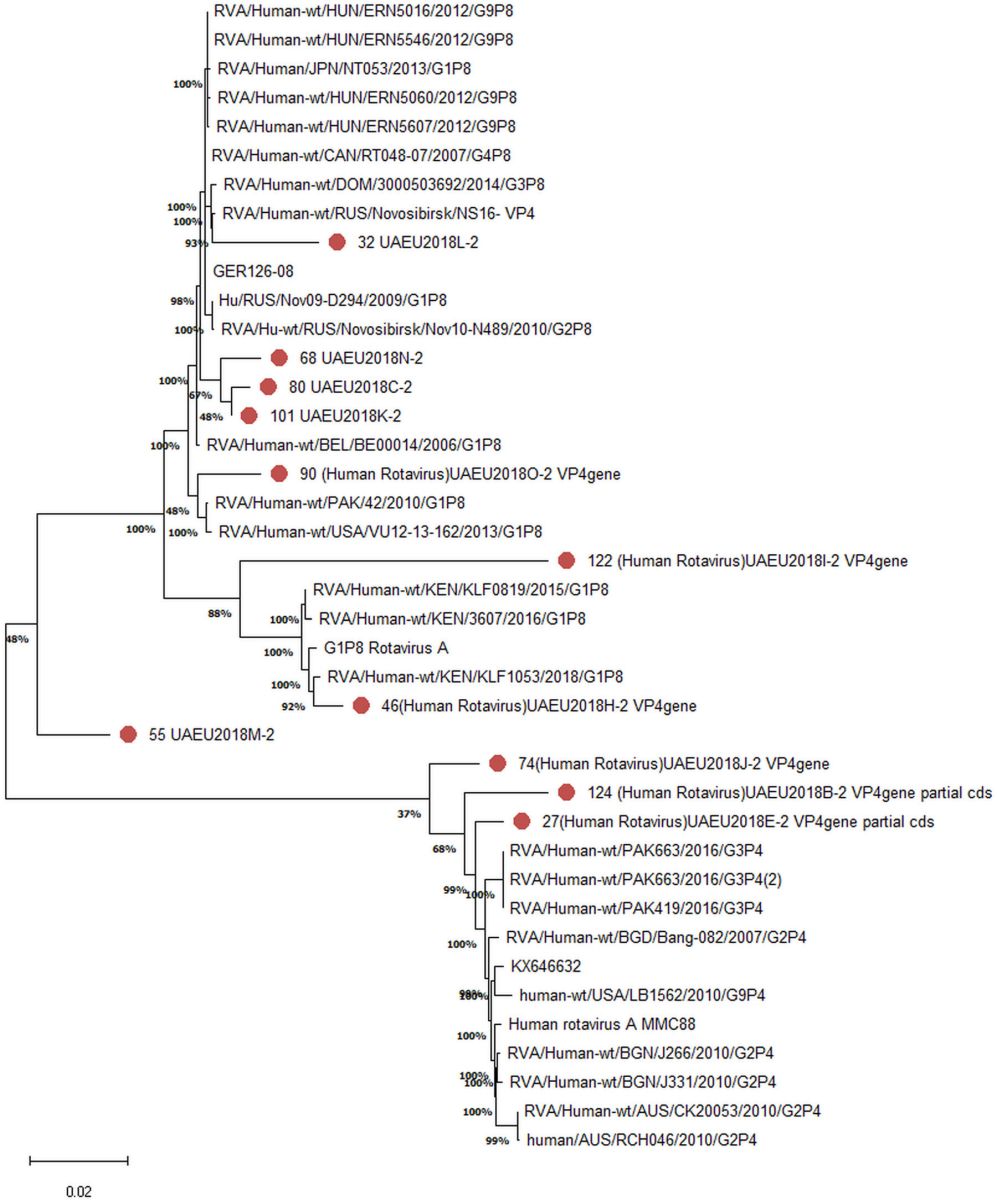
Phylogenetic tree of human rotavirus A strains based on partial VP4 nucleotide sequences. Bootstrap confidence limits are shown at each node; values less than 50 are not shown. Sequences from this study are marked by red color dot.

Based on the obtained partial nucleotide sequences of the VP7 and VP4 genes as well as the sequences of these genes from the GenBank database, multiple alignments were made and phylogenetic dendrograms were plotted separately for each gene (Figure 5 for VP7 and Figure 6 for VP4). Out of 28 cases, only 18 sequences VP7 (GenBank under these accession numbers: ON456079‐ON456085; ON500504‐ON500513) and 11 cases (Accession numbers: ON791605‐ ON791612) were deposited for VP4 genes. Thr remaining Sequences were not submitted to GenBank due to inadequate sequence length.

### 3.3 G Genotypes (VP7)

We have observed three clades for G8 genotype in which UAE isolates were clustered together in one clade. Other clades of G8 were constituted by isolates from India and Europe. It has close similarity with the strains isolated from India. G3 isolates were distributed into two clusters. Among the G3 isolates identified in this study, one was clustered together with Belgian isolate and another with Brazilian isolate. There are two isolates of G3 which were divided into two clades. One isolate was closely resembled with an isolate from Brazil whereas the other isolate shows 100% similarity to an isolate from Belgium. There were five G9 isolates that were identified in this study and were very similar and clustered together, where as a single isolate was found in a different cluster along with isolates from Europe and Africa. There were three clades of G9 genotypes observed in the dendrogram. The UAE isolate were found to be similar to isolates from Pakistan and Iran, but were distant from African isolates. The strain for RotaTeq vaccine belonged to G2 genotype. UAE G2 isolates were found to be more similar to vaccine strain. There were isolates from countries like Argentina, and Bangladesh but were distant from the vaccine strain. Surprisingly, the single isolate for G4 was found to be more closely related to the South American isolate.

Rotarix is the vaccine type being used in the UAE, which belonged to G1 genotype. The two G1 genotypes identified showed close resemblance to the vaccine strain. Using *in silico* analysis, sequences of G1 isolates were compared with Rotarix sequence by pairwise comparison. The isolate #90 showed 97% similarity at nucleotide and amino acid level whereas isolate #94 had 93.87% at nucleotide level and 94.48% at amino acid level. Both UAE strains from this study showed amino acid changes at positions 28 (R‐Q), 41(Y‐S), 65 (T‐A), 66 (V‐A) and 202 (M‐T). The isolate #94 had additional changes in 242(T‐K), 266 (S‐P), 284(M‐I) and 318 (N‐D) positions, Table 1.

**Table 1:** A. Amino acid substitutions observed in UAE RVA strains G1P[8] compared to vaccine strain (Rotarix); B. Percent of nucleotide and amino acid identities of VP7 genes of UAE strains in comparison to Rotarix vaccine strain.

AA. Amino acid positions	28	41	65	66	202	242	266	284	318
Rotarix (JN849114)	R	Y	T	V	M	T	S	M	N
**90 UAEU2018O**	Q	S	A	A	T	T	S	M	N
**94 UAEU2018P**	Q	S	A	A	T	K	P	I	D

BB. Nucleotide identity % (Amino acid identity %)	
**90 UAEU2018O**	**97% (97%)**
**94 UAEU2018P**	**93.87% (94.48%)**

### 3.4 P Genotypes (VP4)

Of the 11 sequences subjected to phylogenic analysis, 8 strains belonged to P8 genotype. The isolates of the present study did not show close similarity with each other's. Isolates #68, 80 and 81 were similar and formed a single cluster. UAE strains were in separate clusters. One isolate was very similar to Eastern Europe strains (#32). Isolate #90 showed similarity with an isolate from Pakistan. Another three isolates were belonging to the P4 genotype which showed similarity to strains from Pakistan and Bangladesh. Isolate number 46 showed close similarity with African isolates. Isolate #55 and 122 were very dissimilar to other isolates and were in distinct branches.

### 3.5 Genotypes and Vaccination History

Out of 27 genotyped rotavirus specimens, 22 were from patients who had received Rotarix vaccine while 3 patients were unvaccinated, one patient had unknown vaccination status and another patient received a vaccine which was unknown, Table 2. Rotavirus genotypes identified from Rotarix‐vaccinated patients were G3P8, G2P4, G8P8, G12P6, G9P4, G4P8 and G9P8. Of these genotypes, G3P8 was the most frequently detected genotype among vaccinated patients, (29%).

**Table 2:** Rotavirus vaccination history.

Sample ID#	Genotype	Vaccination status
16	G9P4	Monovalent (Rotarix)
50	G3P8	Monovalent (Rotarix)
68	G9P8	Monovalent (Rotarix)
74	G2P4	Monovalent (Rotarix)
123	G8P8	Monovalent (Rotarix)
55	G9P8	Monovalent (Rotarix)
90	G1P8	Monovalent (Rotarix)
27	G2P4	No vaccination record
30	G9P4	Unvaccinated
122	G8P8	Monovalent (Rotarix)
10	G12P6	Monovalent (Rotarix)
46	G3P8	Unvaccinated
32	G4P8	Monovalent (Rotarix)
45	G3P8	Monovalent (Rotarix)
124	G12P4	Unvaccinated
168	G8P8	Monovalent (Rotarix)
253	G2P4	Monovalent (Rotarix)
151	G3P8	Monovalent (Rotarix)
173	G8P8	Monovalent (Rotarix)
101	G3P8	Monovalent (Rotarix)
181	G8P8	Monovalent (Rotarix)
93	G9P8	Monovalent (Rotarix)
94	G1P8	Vaccinated (vaccine type is not known)
72	G3P8	Monovalent (Rotarix)
80	G9P8	Monovalent (Rotarix)
97	G3P8	Monovalent (Rotarix)
263	G3P8	Monovalent (Rotarix)

## 4 Discussion

Here, we are reporting the surveillance of rotavirus genotypes from a cohort of children having diarrhea from Al Ain city in the UAE. VP4 and VP7 genes encoding the outer capsid proteins were genetically characterized by two different methods, qRT‐PCR and nanopore sequencing.

Using the simplex TaqMan PCR method, we were able to successfully genotype 16 cases of rotavirus among 28 samples (57.1%). Nanopore sequencing was able to type a total of 28 samples including 15 which could not be typed by RT‐PCR (100%). The genotype G4 was detected only with nanopore sequencing which might be due to changes in the primer/probe binding site in the virus adversely affecting sensitivity of the qRT‐PCR. Interestingly, there was disagreement in the genotypes determined by qRT‐PCR and nanopore sequencing. One isolate detected as G3 by RT‐PCR was determined by nanopore as G9, and another isolate detected as G9 was found to be G3 by nanopore sequencing. There was one nontypeable strain by nanopore sequencing which may be due to the quality and concentration of RNA which are of critical importance. Another possibility, is that the nontypeable strain belongs to a novel genotype, not reported in the previous studies. This may possibly be due to the nature of RVA, an RNA virus known for genetic instability [20]. Moreover, the differences observed in genotyping results may stem from shared sequences in the primers and probes used in qRT‐PCR. While qPCR focuses solely on these specific sequences, nanopore sequencing (NGS) provides a more complete picture of the entire genetic material, allowing for greater differentiation between isolates. Therefore, we consider genotype assignments made using NGS to be more reliable, as they are based on a broader and more detailed data set.

Phylogenetic analysis of VP4 and VP7 genes revealed distinct clustering compared to the vaccine strains, and several substitutions were identified in the antigenic epitopes of UAE specimens, as was reported in a study from the Middle East [21].

The use of nanopore technology for genotyping of RVA was recommended by other investigators who confirmed that the technology can generate both high quality and accurate RVA whole genome sequences from genomic material extracted from fecal samples. Accuracy of nanopore sequencing in RVA genotyping was determined by comparing the results generated by Sanger sequencing against corresponding nanopore sequences demonstrating an overall identity of 100.0% ± 0.02% [22].

In a previous study from the UAE conducted in 2012, before the introduction of the rotavirus vaccine in the country, G1P8 was the most commonly detected RVA strain (56.3%) [14]. However, in our current study, conducted few years later, the prevalence of the G1P8 genotype was only 7%, which is a dramatic shift in the genotype hierarchy happened over the years. Moreover, we found diverse genotypes; among them G3P8 which was found to be more prevalent, followed by G8P8 and G9P8, confirming a drastic change from the previous report.

There are reports showing similar reduction in the prevalence of the G1P8 genotype in US, Pakistan, Kenya and India [23, 24, 25, 26]. However, RV strain distribution has been shown to vary greatly among countries. For example, in Iran, G4P8 is the most predominant RV strain [27] while a varied RV strain distribution is observed in East Asian countries [26, 28, 29, 30]. The detection of uncommon genotypes is an indication that children probably acquire rotavirus infections from various origins, and could become sources of new strains globally. We found no mixed infection in this study, contrary to a review of the epidemiology of rotavirus in India which reported that 9% of rotavirus infections are of mixed type [30].

Moreover, in a study in 1991, epitope mapping using neutralizing monoclonal antibodies identified the amino acids 96, 97 and 100 as neutralizing epitope [31]. The isolates investigated in the present study did not show any changes in these epitopes. This lack of variation suggests that the isolates investigated are not escape mutants, although escape mutant cannot definitely be ruled out.

In this study, a significant number of rotavirus‐positive samples came from vaccinated children, with the G3P8 strain being the most common. While these results may raise concerns, it is important to interpret them carefully. Detection of rotavirus in vaccinated children does not necessarily mean that the vaccine has failed. In fact, vaccination greatly reduces the severity of illness, helping to prevent severe dehydration, hospitalization, and death, even if infection occurs. Several other factors could explain why infections happen in vaccinated individuals, including waning immunity, the emergence of less common strains that are not fully covered by the vaccine, and local epidemiological conditions [32, 33]. The prevalence of the G3P8 strain may simply reflect natural variations in rotavirus genotypes that are less effectively targeted by current vaccines. Furthermore, a comprehensive evaluation of the effectiveness of rotavirus vaccines in the UAE population is currently missing, highlighting the necessity for future research in this area.

Our study expands on the limited knowledge of the genomic epidemiology of rotavirus associated diarrhea in children in the UAE and the region. Rotavirus surveillance and typing are critical, before and during the implementation of any vaccination strategy, as protection from infection and severe disease is dependent on the genotype of vaccine and the circulating strains. There are several distinct genotypes of RVA, which vary in their ability to provide cross‐protection against each other. As the relative prevalence in a population is a dynamic one, ascertaining the genetic profile of circulating viruses and the flux in the relative dominance of the genotypes is of utmost importance to direct the vaccination protocols for effectively controlling the rotavirus epidemic [34].

The present study has few limitations. While our data offer valuable insights into the circulation and dynamics of rotavirus strains in this area, the limited sample may affect the strength of our conclusions. Including a larger and more diverse group of samples from multiple regions across the UAE would enhance the generalizability of our findings. This broader approach would provide a clearer understanding of rotavirus epidemiology in the country and make our conclusions more applicable to the wider population. Also, recent travel history was lacking. Another limitation associated with MinION nanopore sequencing is the requirement of high quality of nucleic acid extracted from the samples. Also, it is important to mention the need for a standardized protocol to get rid of the human genomic materials. Thus, the precipitation protocol was required to improve the yield to have high quality data. Here, we reported for the first time the use of LiCl precipitation to improve nanopore sequencing yield. Based on our results, this method might be useful for other researchers.

## 5 Conclusion

Rotavirus genotype prevalence and hierarchy varies temporally based on several factors such as vaccination history and travel. Such observations emphasize the importance of long‐term surveillance to monitor vaccine impact. The observed changes may represent natural secular variation or possible immuno‐epidemiological changes induced by vaccine. Sequencing based genotyping provides insight into vaccine‐induced antigenic diversity. Here, we show changes in the genotype profile of RVA population circulating in the UAE after the introduction of vaccine. The findings call for a larger study investigating a larger population of the UAE to determine the required changes in the vaccine strategy in the country.

## Author Contributions

**Junu A. George:** investigation, formal analysis, writing original draft, review and editing. **Farah Al‐Marzooq:** formal analysis, review and editing. **Hassib Narchi:** conceptualization, formal analysis, review and editing. **Ahmed R. Alsuwaidi:** conceptualization, resources, formal analysis, supervision, writing–review and editing.

## Ethics Statement

Ethics approval was granted by the Tawam Hospital Human Research Ethics Committee (T‐HREC‐535). Written informed consents were obtained from the parents of the participating children.

## Conflicts of Interest

The authors declare no conflict of interest.

## Data Availability

Data are available on reasonable request to the corresponding author and appropriate ethical approvals (Ahmed R. Alsuwaidi; alsuwaidia@uaeu.ac.ae).

## References

[1] J. E. Tate , A. H. Burton , C. Boschi‐Pinto , and U. D. Parashar , “Global, Regional, and National Estimates of Rotavirus Mortality in Children <5 Years of Age, 2000–2013,” Clinical Infectious Diseases 62, no. suppl 2 (2016): S96–S105, 10.1093/cid/civ1013. 27059362 PMC11979873

[2] World Health Organization , “Rotavirus Vaccines: An Update,” Releve Epidemiologique Hebdomadaire 84, no. 50 (2009): 533–540. 20034143

[3] N. Aliabadi , S. Antoni , J. M. Mwenda , et al., “Global Impact of Rotavirus Vaccine Introduction on Rotavirus Hospitalisations Among Children under 5 Years of Age, 2008‐16: Findings from the Global Rotavirus Surveillance Network,” The Lancet Global Health 7, no. 7 (2019): e893–e903, 10.1016/S2214-109X(19)30207-4. 31200889 PMC7336990

[4] B. D. Hallowell , U. D. Parashar , A. Curns , N. P. DeGroote , and J. E. Tate , “Trends in the Laboratory Detection of Rotavirus Before and After Implementation of Routine Rotavirus Vaccination ‐ United States, 2000‐2018,” Morbidity and Mortality Weekly Report 68, no. 24 (2019): 539–543, 10.15585/mmwr.mm6824a2. 31220058 PMC6586368

[5] World Health Organization . Rotavirus Vaccines: WHO Position Paper ‐ July 2021, accessed October 7, 2024, https://www.who.int/publications/i/item/WHO-WER9628.

[6] J. Matthijnssens , J. Bilcke , M. Ciarlet , et al., “Rotavirus Disease and Vaccination: Impact on Genotype Diversity,” Future Microbiology 4, no. 10 (2009): 1303–1316, 10.2217/fmb.09.96. 19995190

[7] J. Matthijnssens , M. Ciarlet , M. Rahman , et al., “Recommendations for the Classification of Group A Rotaviruses Using All 11 Genomic RNA Segments,” Archives of Virology 153, no. 8 (2008): 1621–1629, 10.1007/s00705-008-0155-1. 18604469 PMC2556306

[8] World Health Organization . Rotavirus: Vaccine Preventable Diseases Surveillance Standards, accessed September 9, 2024, https://www.who.int/publications/m/item/vaccine-preventable-diseases-surveillance-standards-rotavirus.

[9] W. Whitford , V. Hawkins , K. S. Moodley , et al., “Proof of Concept for Multiplex Amplicon Sequencing for Mutation Identification Using the Minion Nanopore Sequencer,” Scientific Reports 12, no. 1 (2022): 8572, 10.1038/s41598-022-12613-7. 35595858 PMC9122479

[10] J. King , T. Harder , M. Beer , and A. Pohlmann , “Rapid Multiplex Minion Nanopore Sequencing Workflow for Influenza A Viruses,” BMC Infectious Diseases 20, no. 1 (2020): 648, 10.1186/s12879-020-05367-y. 32883215 PMC7468549

[11] A. L. Greninger , S. N. Naccache , S. Federman , et al., “Rapid Metagenomic Identification of Viral Pathogens in Clinical Samples by Real‐Time Nanopore Sequencing Analysis,” Genome Medicine 7 (2015): 99, 10.1186/s13073-015-0220-9. 26416663 PMC4587849

[12] Y. Xu , K. Lewandowski , S. Lumley , et al., “Detection of Viral Pathogens With Multiplex Nanopore MinION Sequencing: Be Careful With Cross‐Talk,” Frontiers in Microbiology 9 (2018): 2225, 10.3389/fmicb.2018.02225. 30283430 PMC6156371

[13] Z. Yandle , G. Gonzalez , M. Carr , J. Matthijnssens , and C. De Gascun , “A Viral Metagenomic Protocol for Nanopore Sequencing of Group A Rotavirus,” Journal of Virological Methods 312 (2023): 114664, 10.1016/j.jviromet.2022.114664. 36494024

[14] M. Howidi , N. Al Kaabi , A. C. El Khoury , et al., “Burden of Acute Gastroenteritis Among Children Younger Than 5 Years of Age–A Survey Among Parents in the United Arab Emirates,” BMC Pediatrics 12 (2012): 74, 10.1186/1471-2431-12-74. 22708988 PMC3407526

[15] R. E. Black , J. Perin , D. Yeung , et al., “Estimated Global and Regional Causes of Deaths from Diarrhoea in Children Younger Than 5 Years during 2000‐21: A Systematic Review and Bayesian Multinomial Analysis,” The Lancet Global Health 12, no. 6 (2024): e919–e928, 10.1016/S2214-109X(24)00078-0. 38648812 PMC11099298

[16] A. R. Alsuwaidi , K. Al Dhaheri , S. Al Hamad , et al., “Etiology of Diarrhea by Multiplex Polymerase Chain Reaction Among Young Children in the United Arab Emirates: A Case‐Control Study,” BMC Infectious Diseases 21, no. 1 (2021): 7, 10.1186/s12879-020-05693-1. 33407198 PMC7788778

[17] I. Mwape , S. Bosomprah , J. Mwaba , et al., “Immunogenicity of Rotavirus Vaccine (Rotarixtm) in Infants with Environmental Enteric Dysfunction,” PLoS One 12, no. 12 (2017): e0187761, 10.1371/journal.pone.0187761. 29281659 PMC5744930

[18] R. Gautam , M. D. Esona , S. Mijatovic‐Rustempasic , K. Ian Tam , J. R. Gentsch , and M. D. Bowen , “Real‐Time RT‐PCR Assays to Differentiate Wild‐Type Group A Rotavirus Strains from Rotarix(®) and Rotateq(®) Vaccine Strains in Stool Samples,” Human Vaccines & Immunotherapeutics 10, no. 3 (2014): 767–777, 10.4161/hv.27388. 24342877 PMC4130254

[19] H. Li , “Minimap2: Pairwise Alignment for Nucleotide Sequences,” Bioinformatics 34, no. 18 (2018): 3094–3100, 10.1093/bioinformatics/bty191. 29750242 PMC6137996

[20] J. N. Barr and R. Fearns , “How RNA Viruses Maintain Their Genome Integrity,” Journal of General Virology 91, no. Pt 6 (2010): 1373–1387, 10.1099/vir.0.020818-0. 20335491

[21] H. H. Harastani , L. Reslan , A. Sabra , et al., “Genetic Diversity of Human Rotavirus A Among Hospitalized Children Under‐5 Years in Lebanon,” Frontiers in Immunology 11 (2020): 317, 10.3389/fimmu.2020.00317. 32174920 PMC7054381

[22] E. Faizuloev , R. Mintaev , O. Petrusha , et al., “New Approach of Genetic Characterization of Group A Rotaviruses by the Nanopore Sequencing Method,” Journal of Virological Methods 292 (2021): 114114, 10.1016/j.jviromet.2021.114114. 33662411

[23] A. F. Dennis , S. M. McDonald , D. C. Payne , et al., “Molecular Epidemiology of Contemporary G2P[4] Human Rotaviruses Cocirculating in a Single U.S. Community: Footprints of a Globally Transitioning Genotype,” Journal of Virology 88, no. 7 (2014): 3789–3801, 10.1128/JVI.03516-13. 24429371 PMC3993531

[24] M. Siddiqui , D. A. Salmon , and S. B. Omer , “Epidemiology of Vaccine Hesitancy in the United States,” Human Vaccines & Immunotherapeutics 9, no. 12 (2013): 2643–2648, 10.4161/hv.27243. 24247148 PMC4162046

[25] M. J. Mwanga , B. E. Owor , J. B. Ochieng , et al., “Rotavirus Group A Genotype Circulation Patterns Across Kenya before and after Nationwide Vaccine Introduction, 2010‐2018,” BMC Infectious Diseases 20, no. 1 (2020): 504, 10.1186/s12879-020-05230-0. 32660437 PMC7359451

[26] S. Giri , C. P. G. Kumar , S. A. Khakha , et al., “Diversity of Rotavirus Genotypes Circulating in Children &5 Years of Age Hospitalized for Acute Gastroenteritis in India from 2005 to 2016: Analysis of Temporal and Regional Genotype Variation,” BMC Infectious Diseases 20, no. 1 (2020): 740, 10.1186/s12879-020-05448-y. 33036575 PMC7547507

[27] A. Eesteghamati , M. Gouya , A. Keshtkar , et al., “Sentinel Hospital‐Based Surveillance of Rotavirus Diarrhea in Iran,” The Journal of Infectious Diseases 200, no. Suppl 1 (2009): S244–S247, 10.1086/605050. 19821714

[28] X. Kuang , X. Gong , X. Zhang , H. Pan , and Z. Teng , “Genetic Diversity of Group A Rotavirus in Acute Gastroenteritis Outpatients in Shanghai from 2017 to 2018,” BMC Infectious Diseases 20, no. 1 (2020): 596, 10.1186/s12879-020-05279-x. 32787859 PMC7425067

[29] R. M. Wahyuni , T. Utsumi , Z. Dinana , et al., “Prevalence and Distribution of Rotavirus Genotypes Among Children With Acute Gastroenteritis in Areas Other Than Java Island, Indonesia, 2016‐2018,” Frontiers in Microbiology 12 (2021): 672837, 10.3389/fmicb.2021.672837. 34025628 PMC8137317

[30] C. P. Girish Kumar , S. Giri , M. Chawla‐Sarkar , et al., “Epidemiology of Rotavirus Diarrhea Among Children Less Than 5 Years Hospitalized with Acute Gastroenteritis Prior to Rotavirus Vaccine Introduction in India,” Vaccine 38, no. 51 (2020): 8154–8160, 10.1016/j.vaccine.2020.10.084. 33168345 PMC7694878

[31] N. Kobayashi , K. Taniguchi , T. Urasawa , and S. Urasawa , “Analysis of the Neutralization Epitopes on Human Rotavirus VP7 Recognized By Monotype‐Specific Monoclonal Antibodies,” Journal of General Virology 72, no. Pt 8 (1991): 1855–1861, 10.1099/0022-1317-72-8-1855. 1714939

[32] E. Burnett , U. D. Parashar , and J. E. Tate , “Global Impact of Rotavirus Vaccination on Diarrhea Hospitalizations and Deaths Among Children & 5 Years Old: 2006‐2019,” The Journal of Infectious Diseases 222, no. 10 (2020): 1731–1739, 10.1093/infdis/jiaa081. 32095831 PMC7483971

[33] E. Burnett , U. D. Parashar , and J. E. Tate , “Real‐World Effectiveness of Rotavirus Vaccines, 2006‐19: A Literature Review and Meta‐Analysis,” The Lancet Global Health 8, no. 9 (2020): e1195–e1202, 10.1016/S2214-109X(20)30262-X. 32827481 PMC8097518

[34] M. Iturriza‐Gómara , G. Kang , and J. Gray , “Rotavirus Genotyping: Keeping up with an Evolving Population of Human Rotaviruses,” Journal of Clinical Virology 31, no. 4 (2004): 259–265, 10.1016/j.jcv.2004.04.009. 15494266

